# Impact of an Underlying 2DEG on the Performance of a p-Channel MOSFET in GaN

**DOI:** 10.1021/acsaelm.3c00350

**Published:** 2023-06-08

**Authors:** Jinggui Zhou, Huy-Binh Do, Maria Merlyne De Souza

**Affiliations:** † Department of Electronic and Electrical Engineering, University of Sheffield-Mappin Street, S1 3JD Sheffield, U.K.; ‡ Department of Materials Technology, HCMC University of Technology and Education, 700000 Hochiminh, Vietnam

**Keywords:** 2DHG, E-mode, GaN, MOSFET, p-channel, n-channel, AlGaN, graded channel

## Abstract

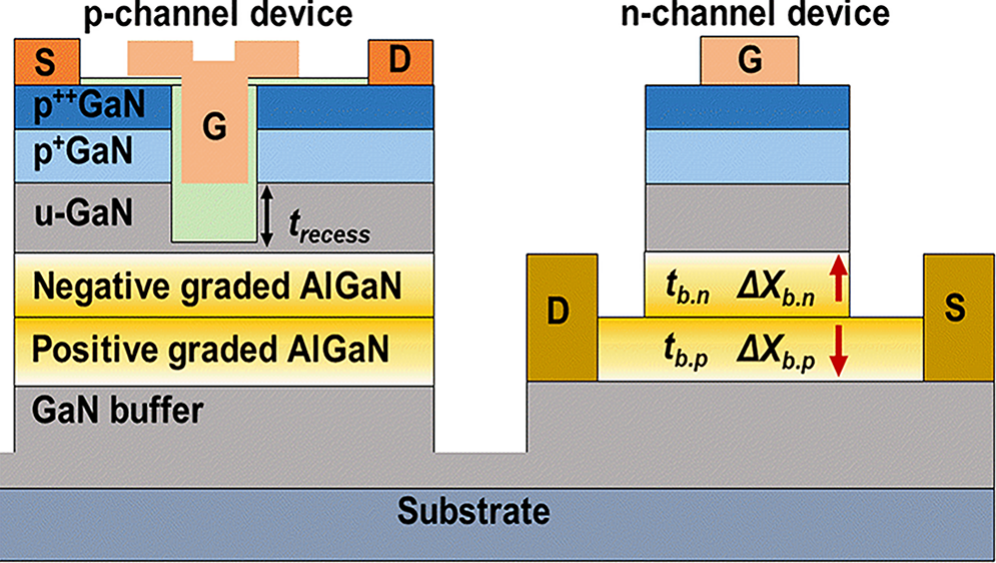

The influence of an underlying 2-dimensional electron gas (2DEG) on the performance of a normally off p-type metal oxide semiconductor field effect transistor (MOSFET) based on GaN/AlGaN/GaN double heterojunction is analyzed via simulations. By reducing the concentration of the 2DEG, a greater potential can be dropped across the GaN channel, resulting in enhanced electrostatic control. Therefore, to minimize the deleterious impact on the on-state performance, a composite graded back-to-back AlGaN barrier that enables a trade-off between n-channel devices and Enhancement-mode (E-mode) p-channel is investigated. In simulations, a scaled p-channel GaN device with *L*_G_ = 200 nm, *L*_SD_ = 600 nm achieves an *I*_ON_ of 65 mA/mm, an increase of 44.4% compared to a device with an AlGaN barrier with fixed Al mole fraction, *I*_ON_/*I*_OFF_ of ∼10^12^, and |*V*_th_| of | – 1.3 V|. For the n-channel device, the back-to-back barrier overcomes the reduction of *I*_ON_ induced by the p-GaN gate resulting in an *I*_ON_ of 860 mA/mm, an increase of 19.7% compared with the counterpart with the conventional barrier with 0.5 V positive *V*_th_ shift.

## Introduction

Gallium nitride (GaN) power devices utilizing a two-dimensional electron gas (2DEG) are promising in applications requiring high voltage, high speed, and low power consumption due to the superior wide-bandgap material properties in comparison with silicon.^1−3^ The monolithic integration of a power device with its gate driver is required to suppress oscillations during high-frequency operation that can irrevocably damage the device.^4^ GaN-based p-channel devices offer the possibility of on-chip complementary logic; however, owing to the low mobility, poor current density, and high resistivity of contacts, such devices are not yet in manufacture.

Several p-channel devices with polarization-induced two-dimensional hole gas (2DHG) at the heterointerface have been reported.^5−10^ Similar to a 2DEG, the 2DHG has characteristics of high density and temperature independence.^11^ However, to achieve a GaN-based complementary metal-oxide-semiconductor (CMOS) technology, p-channel devices with high on-current, enhancement-mode (E-mode), and high on/off ratio are desired to be integrated with a related n-channel power device on the same platform.^7,12−15^ Recently, due to the commercialization of E-mode p-GaN gate high electron mobility power transistors (HEMTs),^1^ p-channel MOSFETs (pFETs) with the same p-GaN/AlGaN/GaN-based epitaxial structure have attracted great interest.^16−19^ Among them, E-mode pFETs with (*I*_ON_/*I*_OFF_) of 3 × 10^8^ and a high threshold voltage (*V*_th_) of | – 2.2 V| were demonstrated.^19^ However, the improved on-current (*I*_ON_) of 18.5 mA/mm by 1.5 nm AlN spacer^19^ is still much lower than that of a generic E-mode n-channel device.^20^ Moreover, in a GaN/AlGaN/GaN-based architecture, an *I*_ON_ of 125 mA/mm was attained at *V*_DS_ = – 20 V for the p-FETs with Schottky gate structure,^21^ but the limitation of leakage in a Schottky gate causes *I*_ON_/*I*_OFF_ < 10^5^. On the other hand, an ultrawide bandgap semiconductor AlN/GaN/AlN-based platform shows potential to increase the current density by maximizing the polarization discontinuity^22−24^ and indicates excellent output power performance in mm-wave integrated circuits,^25,26^ which makes it promising in RF and high-power application. A remarkable *I*_ON_ of 428 mA/mm was obtained in a pFET on this platform,^24^ with an *I*_ON_/*I*_OFF_ of 10^2^ and *V*_th_ of 4 V. Recently, *I*_ON_ > 100 mA/mm and *I*_ON_/*I*_OFF_ > 10^7^ were achieved by a self-aligned gate and FinFET architecture based on the p-GaN/u-GaN/AlGaN/GaN epitaxial structure.^27^ However, normally off operation was achieved by a 40 nm fin width and 50 nm gate recess which can easily convert the device into D-mode if not accurately controlled, as pointed out by the authors. Moreover, a p-GaN/u-GaN/1.5 nm AlN/AlGaN-based p-channel self-aligned FinFET with a fin width of 20 nm was shown to realize an *I*_ON_ of 300 mA/mm but an *I*_ON_/*I*_OFF_ ratio of only 200.^28^ Furthermore, besides gate length, reducing gate width can improve the mobility and current density in GaN HEMTs, and the effective modulation of *I*_ON_, *I*_OFF_, and electric field makes the gate width a key parameter for further optimization of GaN HEMTs.^29^

Considering the commercial maturity of the p-GaN/AlGaN /GaN platform and to avoid impurity scattering at the p-GaN/AlGaN interface, a p-GaN/u-GaN/AlGaN/GaN epitaxial structure, with the potential for monolithic integration without any regrowth, is selected in this work. In addition, the graded AlGaN layer can benefit the linearity and breakdown voltage of GaN HEMTs in RF applications.^30^ We examine the impact of the densities of 2DHG and 2DEG on the performance of a p-channel FET via simulation and explore graded AlGaN barriers to alleviate the tradeoff between the densities of the 2DHG and 2DEG.

## Methodology

**Figure 1:**
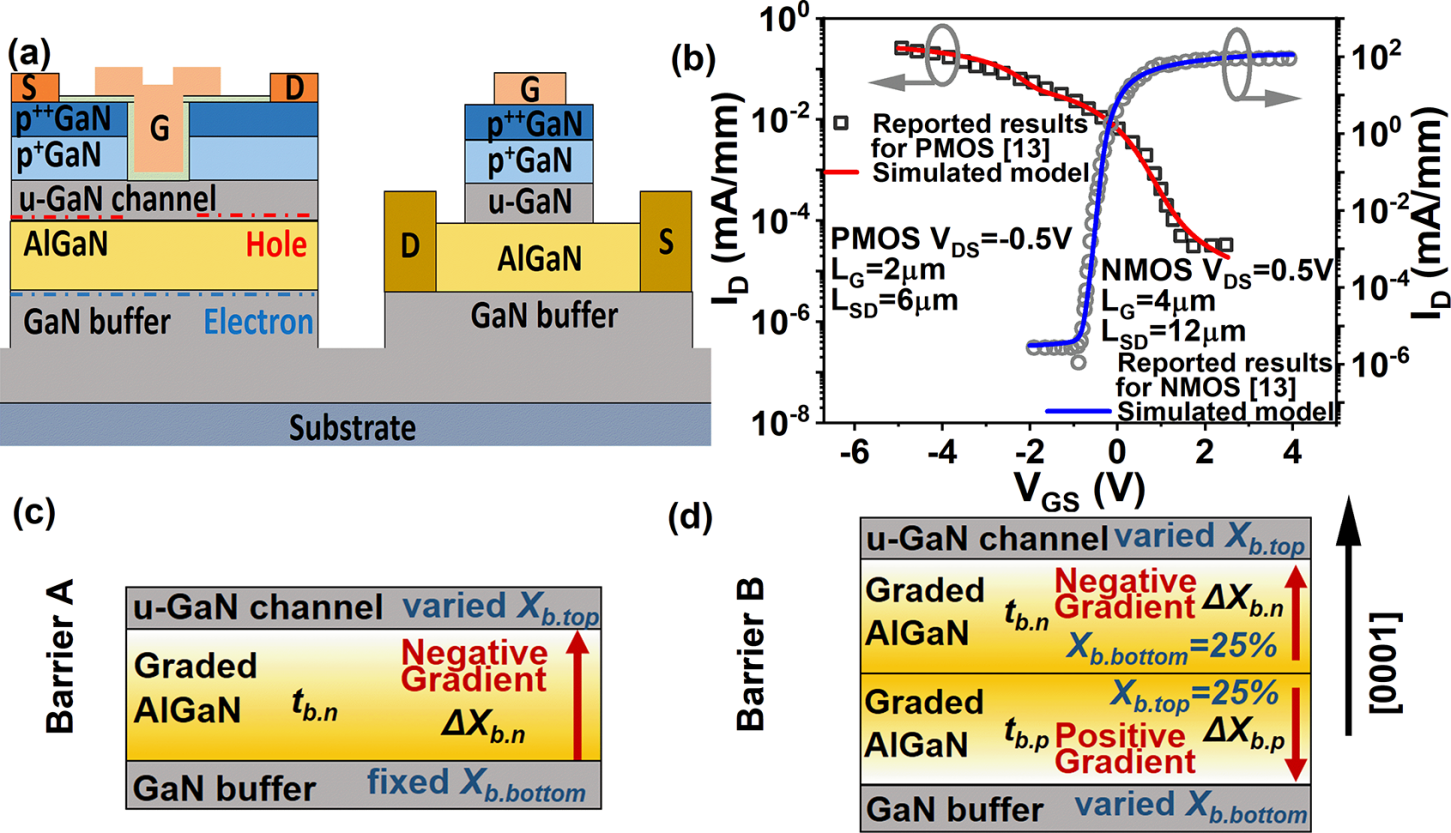
(a) Schematic of the benchmarked p-FET and n-FET from.^13^ (b) Verification of the simulation models with experimental *I*_D_ – *V*_G_ characteristics^13^ of p-FET and n-FET in Silvaco TCAD. Reprinted with permission from ref (13). Copyright [2020][*IEEE Electron Device Lett.*]. The platform structure consists of: (c) Barrier A: A single negatively graded AlGaN. (d). Barrier B: Composite AlGaN, the upper layer is negatively graded AlGaN with *X*_b. bottom_ = 25% on top of a positively graded AlGaN with fixed *X*_b. top_ = 25%.

Figure 1a shows the schematic of the platform consisting from top to bottom of 20 nm of p^++^GaN doped with Mg concentration of 6 × 10^19^ cm^–3^, 50 nm of p^+^GaN (Mg: 1 × 10^19^ cm^–3^), 20 nm of undoped GaN (u-GaN) as a channel layer (*t*_ch_), and 20 nm of Al_0.2_Ga_0.8_N barrier layer (*t*_b_) below which lies a 150 nm-thick u-GaN and buffer layer reported in ref (13). Our simulations are first benchmarked based on the reported epitaxial stack in Silvaco TCAD using a gate length (*L*_G_) of 2 and 4 μm for p-channel and n-channel devices, respectively, reported in ref (13). The source to drain distance (*L*_SD_) is 6 and 12 μm for the p- and n-FET, respectively. In the p-FET, the gate oxide is 20 nm Al_2_O_3_, with the recessed gate etched out of the p^++^GaN and p^+^GaN to support E-mode operation. Based on Hall measurements, a mobility value of 10 cm^2^/Vs of holes in a 2DHG is used.^13^Figure S1 reveals that the mobility of holes in the 3DHG is ∼10.4 cm^2^/Vs and its peak value changes relatively with the density of 3DHG in the graded AlGaN in our simulation model (Supporting Information A). In addition, a negative fixed interface charge and trap density at the oxide/GaN interface of 6.4 × 10^12^ and 3 × 10^12^ cm^–2^, respectively, are introduced in our model to match the reported experimental *I*_D_ – *V*_GS_ curves in Figure 1b.^13^ Two kinds of graded AlGaN barriers are introduced in this work. Along the [0001] growth direction, the negatively graded AlGaN has an Al mole fraction (*X*_b_) that is linearly reduced from the bottom (*X*_b. bottom_, close to the buffer layer) to the top (*X*_b. top_, close to the u-GaN channel), viz., Δ*X*_b. n_ = *X*_b. bottom_ – *X*_b. top_. On the other hand, a positively graded AlGaN has an opposite structure, i.e., Δ*X*_b. p_ = *X*_b. top_ – *X*_b. bottom_. In addition, several new kinds of composite barriers with varied thickness of negatively graded barriers (*t*_b. n_) and positively graded barrier (*t*_b. p_) are listed in Table 1. Barrier A is a single negatively graded AlGaN layer with fixed *X*_b. bottom_ and varied *X*_b. top_ displayed in Figure 1c. Figure 1d exhibits a dual back-to-back graded AlGaN Barrier B, and the upper layer is negatively graded AlGaN with 25% *X*_b. bottom_ and changed *X*_b. top_, whereas the layer beneath is positively graded AlGaN with 25% *X*_b. top_ and varied *X*_b. bottom_. Δ*X*_b. p_ is kept the same as Δ*X*_b. n_. In this work, our assumption of a 4.95% Al/nm gradient^31^ and 5 nm graded AlGaN with 23% Δ*X*_b. p_^32^ make the 5 nm graded AlGaN with 25% gradient to be practically possible. Considering that the p-GaN gate in n-FETs on this platform requires layers to be etched up to the positively graded AlGaN, a precise etch stop at the negative/positively graded AlGaN interface is essential.

**Table 1:** Details of Optimized Barrier Layers

ID	barrier parameters
barrier A	negatively graded AlGaN
barrier A1	*t*_b. n_ = 20 nm, *X*_b. bottom_ = 20%
arrier A2	*t*_b. n_ = 20 nm, *X*_b. bottom_ = 25%

ID	upper layer parameters	lower layer parameters
barrier B	negatively graded AlGaN, *X*_b. bottom_ = 25%	positively graded AlGaN, *X*_b. top_ = 25%
barrier B1	*t*_b. n_ = 5 nm	*t*_b. p_ = 15 nm
barrier B2	*t*_b. n_ = 10 nm	*t*_b. p_ = 10 nm
barrier B3	*t*_b. n_ =1 5 nm	*t*_b. p_ = 5 nm

## Results and Evaluation

**Figure 2:**
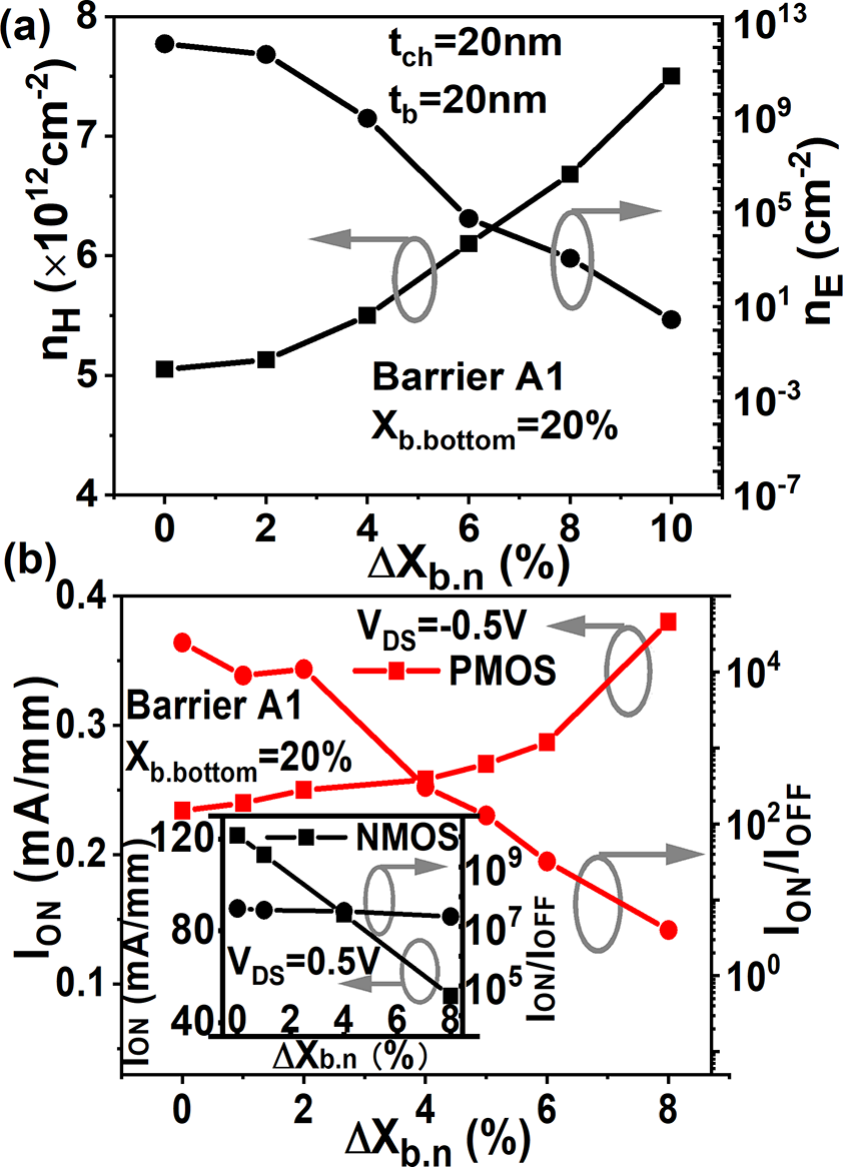
(a) *n*_H_ in p-channel and *n*_E_ in n-channel with respect to a change in Δ*X*_b. n_ based on the platform with Barrier A1. (b) *I*_ON_ and *I*_ON_/*I*_OFF_ as a function of Δ*X*_b. n_ at *V*_DS_= −0.5 V for PMOS with Barrier A1. The inset indicates *I*_ON_ and *I*_ON_/*I*_OFF_ as a function of Δ*X*_b. n_ at *V*_DS_ = 0.5 V for NMOS with Barrier A1.

To examine the influence of the underlying 2DEG density on the performance of p-FETs, the Al_0.2_Ga_0.8_N barrier layer of the epitaxial structure in Figure 1a is replaced by Barrier A1, whereas the effects of a single positively graded AlGaN (counterpart of Barrier A) on both n- and p-channel FETs are shown in Figure S2 (Supporting Information B). Figure 2a reveals that the hole density (*n*_H_) increases as the underlying electron density (*n*_E_) is reduced by an increase of Δ*X*_b. n_. Although a 1.5 times improvement is achieved for *n*_H_ with 10% Δ*X*_b. n_, *n*_E_ reduces dramatically by 12 orders and below a value of 10/cm^2^, which will severely degrade n-channel devices. According to Figure 2b, for Barrier A1, when Δ*X*_b. n_ is increased to 8%, the on-current (|*I*_ON_|) of the PMOS is doubled compared with the standard device with Al_0.2_Ga_0.8_N barrier at *V*_DS_ = – 0.5 V, whereas the on/off ratio (*I*_ON_/*I*_OFF_) reduces from 10^5^ to 10^0^. In contrast, a threefold drop of *I*_ON_ occurs in the n-channel device with Barrier A1 as Δ*X*_b. n_ is raised from 0 to 8% as shown in the inset of Figure 2b, which means the negatively graded barrier unilaterally benefits *I*_ON_ for p-FETs. Moreover, unlike in p-FETs, *I*_ON_/*I*_OFF_ in n-FETs is immune to the changes of Δ*X*_b. n_ and *n*_E_. The reason is because although graded AlGaN barriers redistribute channel carriers, the off-state current (*I*_OFF_) of n-channel devices, in practice, is mainly affected by traps in the buffer layer- or surface-related conduction.^33^

**Figure 3:**
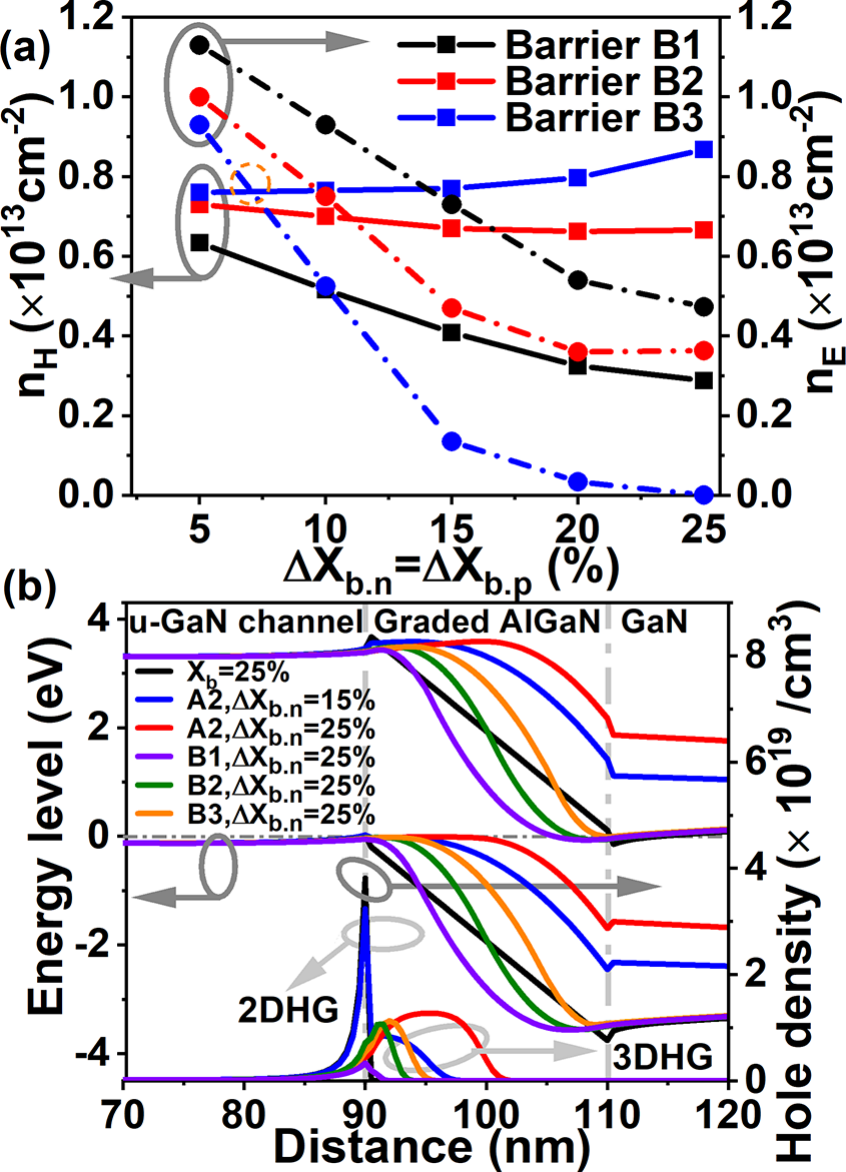
(a) Comparison of *n*_H_ and *n*_E_ as a function of Δ*X*_b. n_ in the composite AlGaN barriers of *t*_b_ = 20 nm that consist of Barriers B1, B2, and B3. (b) Comparison of band diagram and distribution of hole concentration between the devices with fixed Al_0.25_Ga_0.75_N barrier layer, Barrier A2 with 15 and 25% Δ*X*_b. n_ and Barriers B1, B2, and B3 with 25% Δ*X*_b. n_ at *V*_g_ = *V*_d_ = 0 V.

This one-way gain makes the single-graded barrier layer disadvantageous to p- or n-channel devices and complementary integration, and requires an additional positively graded AlGaN below the negatively graded AlGaN layer (Figure 1d) as investigated in Figure 3a. It is obvious from Figure 3a that with Barrier B, both *n*_E_ and *n*_H_ show degradation with increased Δ*X*_b. n(b. p)_ when the thickness of the positively graded AlGaN *t*_b. p_ increases, which means polarization-induced holes and electrons in a back-to-back graded AlGaN with optimized *t*_b. n_, *t*_b. p_, and Δ*X*_b. n(b. p)_ are mutually restricted instead of unilaterally suppressed. In this structure, the optimized tradeoff points between *n*_H_ and *n*_E_ are obtained in Barrier B3 with Δ*X*_b. n(b. p)_ = 7% resulting in a concentration of ∼7.8 × 10^12^cm^–2^for both holes and electrons.

Figure 3b demonstrates that the hole quantum well is weakened by the negatively graded barrier layer at the u-GaN channel/AlGaN barrier interface. When comparing Al_0.25_N_0.75_ with Barrier A2, the larger Δ*X*_b. n_ introduces a curvature of the barrier layer, resulting in the quantum well at the u-GaN/AlGaN junction becoming flatter and wider. It is observed that in Barrier A2, this “flat” quantum well not only accumulates a 2DHG at the u-GaN/AlGaN interface but also induces a three-dimensional hole gas (3DHG) in the barrier layer. It is well known that a graded barrier layer results in polarization doping without impurity dopants, which can improve the conductivity of the graded layer.^34^ In addition, as Δ*X*_b. n_ reaches the maximum value of 25%, the 2DHG at the u-GaN/AlGaN interface in Barrier A2 disappears and only a broad and flat 3DHG distribution in the barrier layer is observed. This 3DHG also broadens the channel width to achieve a higher current density in p-FETs. However, the conduction band at the AlGaN/GaN buffer interface is raised away from the Fermi level in Barrier A2 due to the large Δ*X*_b. n_, which contributes to the potential in the barrier layer being shifted toward negative values and the hole confinement subsequently reduced in Barrier A2. As a result, the ability of the polarization field for blocking punch-through leakage paths into the buffer layer is weakened.

On the other hand, when the positively graded AlGaN is introduced, it is seen from Figure 3b that the conduction band reverts to the Fermi level at *V*_GS_ = 0 V and lies below the Fermi level at the AlGaN/GaN interface with an extension into the AlGaN barrier layer. The band offset in Barrier B with a thicker *t*_b. p_ is larger and sharper. Consequently, the width and peak of the 3DHG across the negatively graded barrier are limited by *t*_b. p_ as shown in Figure 3b. Thus, despite the maximum Δ*X*_b. n_, *n*_H_ still reduces with larger *t*_b. p_ as Figure 3a indicates. Furthermore, Figure 3b illustrates that based on Barriers B2&B3 with 25% Δ*X*_b. n(b. p)_, quantum wells at the GaN channel/AlGaN and AlGaN/GaN buffer interfaces both turn “flat” and are pinned to the Fermi level with a large band offset at the AlGaN/GaN interface. Consequently, the distributions of the potential and polarization-induced charges are rebuilt in the barrier layer with excellent hole confinement in the channel, which is surmised to suppress the leakage in p-channel devices.

**Figure 4:**
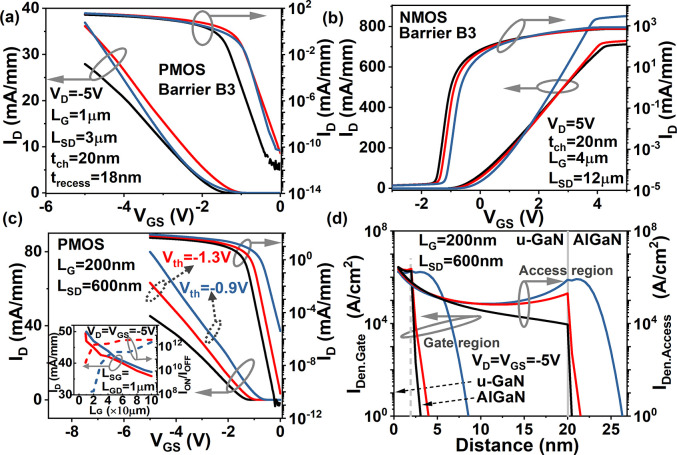
Black line: fixed Al_0.25_Ga_0.75_N barrier layer; Red line: Barrier B3 with 7% Δ*X*_b. n(b. p)_; Blue line: Barrier B3 with 25% Δ*X*_b. n(b. p)_. (a) Comparison of the transfer characteristics in linear and log scale between PMOS with different barriers at *L*_G_ = 1 μm, *L*_SD_ = 3 μm, *t*_recess_ = 18 nm, and *V*_D_ = – 5 V. (b) *I*_D_ – *V*_GS_ characteristics in log and linear scale of n-channel devices based on the same platform as Abstract figure at *L*_G_ = 4 μm, *L*_SD_ = 12 μm, and *V*_D_ = 5 V. (c) Comparison of the *I*_D_ – *V*_GS_ curves in linear and log scale between the scaled PMOS with Al_0.25_Ga_0.75_N and Barrier B3 at *L*_G_ = 200 nm, *L*_SD_ = 600 nm, *t*_recess_ = 18 nm, and *V*_D_ = – 5 V. The inset reveals *I*_D_ and *I*_ON_/*I*_OFF_ at *V*_D_ = *V*_GS_ = – 5 V with a change in gate length with fixed *L*_SG_ = *L*_GD_ = 1 μm for PMOS with Barrier B3. (d) Comparison of current density around the u-GaN channel/AlGaN interface under the gate and access region between PMOS with various barriers at *L*_G_ = 200 nm, *L*_SD_ = 600 nm, and *V*_D_ = *V*_GS_ = – 5 V.

Abstract figure displays the new back-to-back graded AlGaN barrier-based platform for complementary integration. To achieve an E-mode and high *I*_ON_/*I*_OFF_ for PMOS, a gate recess depth in the u-GaN channel layer (*t*_recess_) of 18 nm is used. Moreover, in n-channel devices, to prevent degradation of on-current caused by the reduced *n*_E_ as shown in Figure 3a, the top negative AlGaN is etched away beneath the p-GaN gate. Barrier B3 with a tradeoff of 7% Δ*X*_b. n_ and a maximum of 25% Δ*X*_b. n_ are applied to NMOS with *L*_G_ = 4 μm and scaled PMOS with *L*_G_ = 1 μm as shown in Figure 4a,b. Figure 4a reveals that the on-state current density of p-channel devices with Barrier B3 is increased compared to the counterpart with a fixed Al mole fraction due to the polarization doping and enlarged *n*_H_ by a negatively graded AlGaN. In addition, unlike the deterioration of *I*_ON_/*I*_OFF_ in a single negatively graded AlGaN, *I*_ON_/*I*_OFF_ > 10^12^ with Barrier B3 and the large Δ*X*_b. n_ prove that the positively graded AlGaN improves hole confinement and band offset in the composite AlGaN barrier to suppress the leakage current into the GaN buffer layer in the PMOS in Figure 3b.

At *V*_D_ = *V*_GS_ = 5 V, *I*_ON_ of n-channel devices with Barriers B3 is improved by 19.7% compared with a device with fixed Al mole fraction as shown in Figure 4b. Similar to the role of a negatively graded AlGaN in PMOS, the positively graded AlGaN enhances the channel depth with 3DEG polarization doping and redistributes the potential and electrons in n-channel devices, which means the mutual limitation between the hole and electron channels in the GaN/AlGaN/GaN-based epitaxial platform and the decrease of *I*_ON_ induced by the p-GaN gate in n-channel devices is overcome by the back-to-back graded AlGaN. In addition, n-channel devices with Barrier B3 all reveal a drift of *V*_th_ toward the positive direction with no change in the off-state current level. The reason is that the negatively graded AlGaN in the p-GaN gate region depletes the electrons below and *I*_OFF_ in n-FETs is dominated by surface-related conduction and buffer layer characteristics.^33^ To realize a better gate electrostatic control for n-FETs, the thinner u-GaN channel thickness and fin-gate structure are feasible. Moreover, Figure S3b illustrates the effects of under-etch and over-etch of the p-GaN gate region on the performance of n-FETs (Supporting Information C). The maximum degradation of *I*_ON_ observed is ∼30%. Furthermore, we have shown that the figure of merit (FOM = *V*_BV_^2^/*R*_on, sp_) can be improved by 3 times in a GaN power HEMT with the back-to-back graded AlGaN compared to the one with a conventional Al_0.25_Ga_0.75_N.^35^

The impact of gate length scaling is investigated at *V*_D_ = *V*_GS_ = – 5 V in Figure 4c, via a Barrier B3-based PMOS, with *L*_G_ = 200 nm and *L*_SD_ = 600 nm, which is shown to increase |*I*_ON_| from 45mA/mm, for a fixed Al_0.25_Ga_0.75_N barrier, to 65mA/mm with large *I*_ON_/*I*_OFF_ of ∼10^12^ and threshold voltage | – *V*_th_| of | – 1.3 V|. A further improvement of |*I*_ON_| of 23% with | – *V*_th_| of | – 0.9 V| with Barrier B3 is possible with 25%Δ*X*_b. n_; however, *I*_ON_/*I*_OFF_ decreases significantly by 5 orders, which indicates that in a shorter gate, it is difficult to deplete holes with a wider channel depth. The inset of Figure 4c illustrates that |*I*_ON_| of the PMOS with Barrier B3 of 7% Δ*X*_b. n_ is increased by 39% when *L*_G_ is reduced from 1 μm to 90 nm with source-to-gate (*L*_SG_) and gate-to-drain (*L*_GD_) length maintained at 1 μm. Meanwhile, *I*_ON_/*I*_OFF_ of the PMOS with Barrier B3 of 7%Δ*X*_b. n_ stays above 10^12^ as *L*_G_ ≥ 200 nm. The inset of Figure 4c also indicates that although the larger hole spread of Barrier B3 of 25%Δ*X*_b. n_ enhances |*I*_ON_| during scaling of *L*_G_, *I*_ON_/*I*_OFF_ is worsened significantly owing to insufficient depletion region across the 3DHG slab.

Figure 4d demonstrates a comparison of the current density distribution from a u-GaN channel to the AlGaN barrier under the gate region (*I*_Den. Gate_) and the access region between the gate and drain (*I*_Den. Access_) in a PMOS with Al_0.25_Ga_0.75_N and Barrier B3 at 200 nm *L*_G_. In Figure 4d, at −5 V of *V*_D_ and *V*_GS_, *I*_Den. Gate_, and *I*_Den. Access_ both achieve a spread distribution across the AlGaN barrier with Barrier B3 with 25% Δ*X*_b. n_-based PMOS, which proves that the extended “flat” quantum well at the u-GaN/AlGaN interface. This occurs because a large gradient leads to holes drifting into the negative barrier layer instead of the limited 2-dimensional transport direction in the abrupt quantum well and also leakage currents are more likely to flow through these areas. Furthermore, 25% Δ*X*_b. n_ induces the widest 3DHG slab across the barrier, which results in an improved barrier conductivity and broadened current flow path that is difficult to be depleted and controlled by a short gate. Therefore, the leakage current is more controllable in Barrier B3 with 7% Δ*X*_b. n_ during gate scaling. Utilizing a fin gate configuration has the potential to suppress the leakage current and take full advantage of the back-to-back graded AlGaN barrier with large gradient.

## Conclusions

This work provides a new back-to-back graded barrier platform for GaN-based CMOS. It is seen that the underlying 2DEG of PMOS is lowered by increasing Δ*X*_b. n_ in the negatively graded AlGaN buffer layer, which results in a significantly higher on-current density. A recess gate E-mode p-channel MOSFET with *L*_G_ = 200 nm and *t*_recess_ = 18 nm demonstrates |*I*_ON_| = 65mA/mm, *I*_ON_/*I*_OFF_ ≈ 10^12^ and | – *V*_th_| = | – 1.3 V| is realized by the back-to-back graded barrier with an optimum 7% Δ*X*_b. n(b. p)_. Furthermore, for the back-to-back graded AlGaN barrier layer, not only does the negatively graded AlGaN on the top result in high current density and conductivity in p-channel devices but also the positively graded AlGaN below contributes to a rise of the on-state current by 19.7% in n-channel devices.

## Supporting Information Available

Mobility model for 2DHG and 3DHG; effects of single positively graded barrier on n-FETs and p-FETs; influences of the under-etch and over-etch for p-GaN gate region on n-FETs (PDF)
