# Supplementary material for: Impact of an Underlying 2DEG on the Performance of a p-Channel MOSFET in GaN
Source: ACS Appl Electron Mater. 2023 Jun 8;5(6):3309–15. doi: 10.1021/acsaelm.3c00350 (PMC10308811; doi:10.1021/acsaelm.3c00350)
Supplement: Supplementary file 1 — el3c00350_si_001.pdf [file el3c00350_si_001.pdf]

Supporting information:

The impact of an underlying 2DEG on the performance of a p-channel MOSFET in GaN

Jinggui Zhou[†], Huy-Binh Do [††], Maria Merlyne De Souza [*, †].

[†]Department of Electronic and Electrical Engineering, University of Sheffield-Mappin Street, S1 3JD, Sheffield, UK.

[††]Department of Materials Technology, HCMC University of Technology and Education, 700000, Hochiminh, Vietnam.

Corresponding Author

* m.desouza@sheffield.ac.uk.

A. The mobility model in TCAD simulations

The hole mobility model in this work is calibrated by combining the Albrecht model for low field and the nitride specific field dependent mobility model for high field in Silvaco TCAD [1]. The 2DHG mobility in the model is set as $10cm^2/V.s$, and the mobility model is doping concentration and electric field dependent, so the carrier mobility changes accordingly based on the changing density and electric field distribution induced by the graded layer shown in Figure S1. The mobility of a 3DHG is improved a little to $\sim 10.4cm^2/V.s$. And as the 3DHG width widens due to an increasingly negative gradient ($\Delta X_{b.n}$), the distribution of the peak mobility is relatively broadened in the 3DHG.

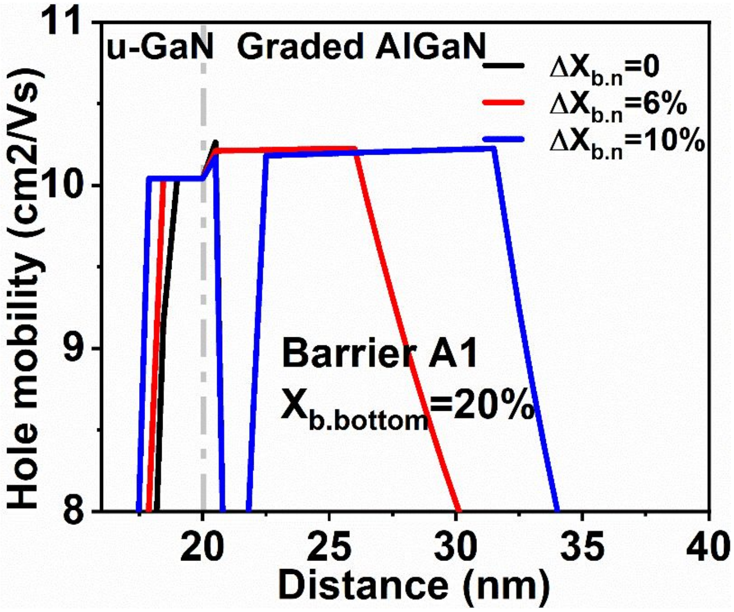

Figure S1. A comparison of the distribution of hole mobility in a Barrier A1-based platform with different $\Delta X_{b.n}$.

B. The influence of single positively graded AlGaN barrier on n- and p-FETs

It is seen from Figure.S2, that a positively graded AlGaN layer exhibits the exact opposite effect on n- and p-channel FETs compared to a negatively graded AlGaN. As the $X_{b.top}$ of the positively graded AlGaN is pinned at 20%, the positive gradient ($\Delta X_{b.p}$) of 10% contributes to 1.5 times improvement in the underlying electron density (n_E), which results in a 47% decrease of hole density (n_H) shown in Figure.S2(a). Figure.S2(b) indicates that as opposed to the negatively graded AlGaN, 8% $\Delta X_{b.p}$ leads to a reduction of on-current (I_{ON}) by 14.5% and increase by 16.5% for p-FETs and n-FETs, respectively. Moreover, on/off current ratio (I_{ON}/I_{OFF}) of p-FETs is significantly increased from 10^5 to 10^{11}, whereas on the contrary, that of n-FETs still shows negligible change based on a positively graded barrier. I_{ON} and n_H in p-FETs tend to saturate with positively graded barrier at large $\Delta X_{b.p}$. The reason is that the hole density at the u-GaN/AlGaN interface is considerably reduced with large $\Delta X_{b.p}$. The majority carriers in p-FETs are dominated by holes in the p-GaN layer.

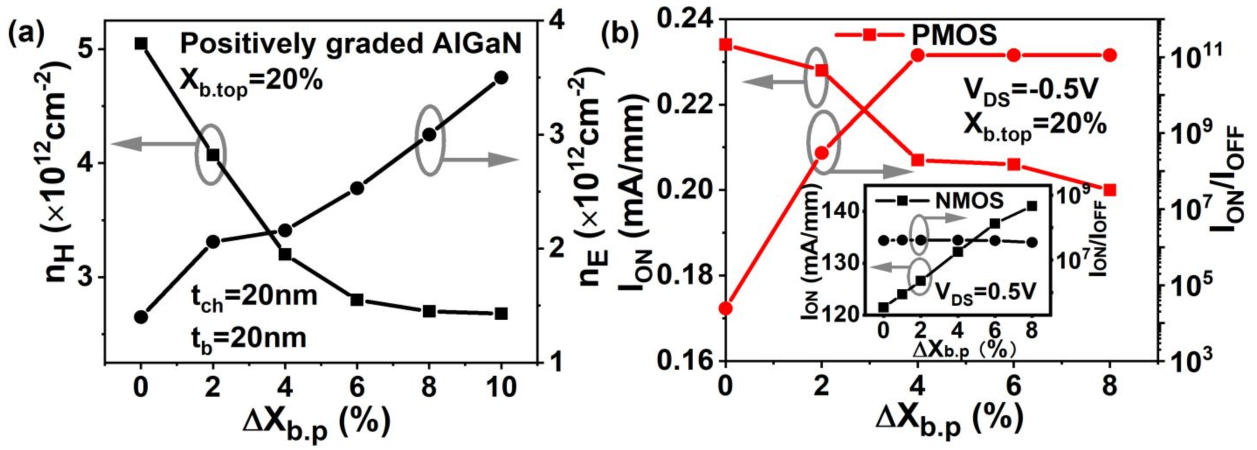

Figure S2. (a) n_H in p-channel and underlying n_E in n-channel with respect to a change in $\Delta X_{b.p}$ based on the platform with positively graded AlGaN barrier. (b) I_{ON} and I_{ON}/I_{OFF} as a function of $\Delta X_{b.p}$ at V_{DS}=-0.5V for PMOS with positively graded AlGaN. The inset indicates I_{ON} and I_{ON}/I_{OFF} as a function of $\Delta X_{b.p}$ at V_{DS}=0.5V for NMOS with positively graded AlGaN.

C. The effects of under-etch and over-etch of the p-GaN gate region on the performance of n-FETs

The precision of etch stop at the negatively graded AlGaN/positively graded AlGaN interface is a challenge to achieve a p-GaN gate of the n-FETs. To analyze the effect of imprecise etching on the device performance, an unexpected layer thickness defined as t_{remain} is illustrated in Figure S3(a). During under-etch ie a part of the negatively graded AlGaN remains, t_{remain} is < 0. Oppositely, $t_{remain} > 0$ represents over-etch causing a part of the positively graded AlGaN to be additionally etched. Figure S3(b) reveals that as long as $|t_{remain}| > 0$, n-FETs will suffer from degradation of I_{ON} and the maximum reduction for I_{ON} is ~30%. In addition, if the etch depth tolerance is controlled within ± 2nm, the decrease of I_{ON} can be limited to around 2.4%. Moreover, according to a comparison of the transfer curves in log scale in the inset of Figure S3(b), the under-etch and over-etch have no influence on the I_{ON}/I_{OFF} and threshold voltage of n-FETs in the ideal simulation condition due to the unchanged depletion region under the p-GaN gate and characteristics of buffer layer.

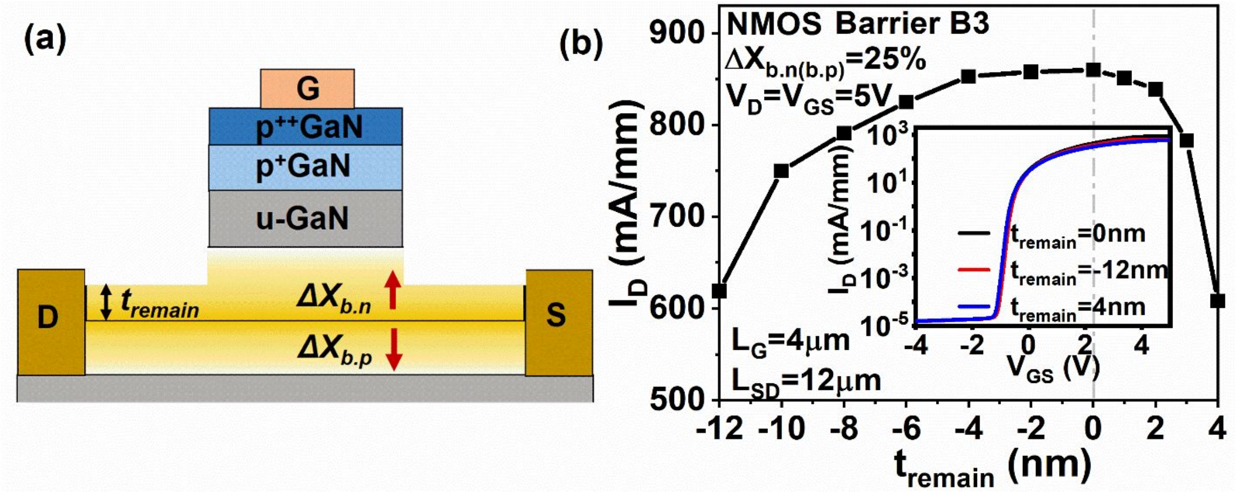

Figure S3. (a) A schematic cross section of the p-GaN gate n-FET with under-etch or over-etch. (b) I_{ON} vs. t_{remain} for the Barrier B3 based n-FET with 25% $\Delta X_{b.n(b.p)}$, $L_G = 4\mu m$, $L_{SD} = 12\mu m$ and at $V_D = V_{GS} = 5V$. The inset shows a comparison of $I_D - V_{GS}$ curves on a log scale between n-FETs with different t_{remain}.

References

(1) *Silvaco TCAD Atlas*. https://silvaco.com/tcad/ for an overview of its features such as device simulation (last accessed April 27, 2023).
